# Editorial: Period poverty

**DOI:** 10.3389/frph.2023.1140981

**Published:** 2023-02-07

**Authors:** Lea Sacca, Christine Margaret Markham, Jhumka Gupta, Melissa Peskin

**Affiliations:** ^1^ Charles E. Schmidt College of Medicine, Florida Atlantic University, Boca Raton, FL, United States; ^2^ Center for Health Promotion and Prevention Research, School of Public Health, University of Texas Health Science Center Houston, Houston, TX, United States; ^3^ Department of Global and Community Health, College of Health and Human Services, George Mason University, Fairfax, VA, United States

**Keywords:** period poverty, menstrual equity, women's rights, sanitary facilities, menstrual hygiene, menstrual health, human rights

**Editorial on the Research Topic** Period poverty

Each month, half of the world's female population, estimated at 1.9 billion individuals, experience menstruation (1). Yet, as normal as it seems, menstruation is still stigmatized around the world, particularly in low-income countries with inadequate access to menstrual health and hygiene management resources (1, 2). Menstrual health is a critical global public health issue that has been impacting the physical, mental, and socioeconomic health of both women and girls (2). However, there still exists social, cultural, economic, and political barriers to accessing menstrual products, menstruation educational material, and sanitation facilities when needed (3, 4).

The American Medical Women's Association defines period poverty as the lack of accessibility or affordability of menstrual hygiene tools and educational material, such as sanitary products, washing facilities, and waste management (5). The term also refers to the increased economic vulnerability menstruating people face due to the financial burden posed by menstrual supplies, which are not only limited to menstrual pads and tampons, but also include costs accrued from pain medication and underwear used during the menstruation cycle (6–8). Such resource-limited settings drive women and girls to improvise with unsanitary alternatives such as old blankets, chicken feathers, old rags, newspapers, and cow dung (4). Difficulty affording menstrual products can cause women and girls to stay home from school and work, with lasting consequences on their education and economic opportunities. It can also exacerbate existing vulnerabilities, pushing individuals closer toward dangerous coping mechanisms (9, 10).

Period poverty does not only affect menstruating people in lower income countries; it also affects them in higher income countries (11). It is estimated that 16.9 million menstruating women in the United States live in poverty, two-thirds of which are low-income and food-insecure women who cannot afford basic menstrual products such as pads, tampons, and menstrual products. One national survey among college students found that 14.2% of respondents reported experiencing period poverty in the year preceding the survey, while an additional 10% experienced it every month (12). The same study documented period poverty being associated with higher likelihood of symptoms consistent with moderate/severe depression (12), with those reporting monthly period poverty at greatest risk for reporting such symptoms. Period poverty has also been associated with mental health issues and urinary tract infections (3, 4).

The gap in the literature and the lack of data on period poverty is a continuous challenge to measure its deteriorating impact on women's health. The following special issue aimed to address this gap by including a diverse set of qualitative, cross-sectional, and review studies exploring the menstrual experiences of girls and women from different regions across the world. For instance, the menstrual health challenges reported by adolescent girls in Myanmar ranged from access to information and social support, to behavioral restrictions, stigma surrounding menstruation, and difficulties managing menstrual bleeding and pain (Swe et al.). These findings were similar to those highlighted in the systematic review by Patel et al. on period poverty in lower and middle income countries, which included three emerging themes:

1. Availability and affordability of menstrual products, and accessibility to water, sanitation and health (WASH) services,
2. Availability of support system and coping with “period poverty,” and
3. Gender dimensions of menstrual hygiene management.

Age appropriate education, using innovative approaches such as interpersonal practical guidance (IPG) as face-to-face communication and mediated practical guidance (MPG) as social and behavior change communication (SBCC), was successful in improving menstrual health and hygiene management (MHHM) knowledge, attitudes, and practices in India, while addressing the existing menstrual health challenges in similar settings (Block et al.). Another country that suffered drastically from the consequences of period poverty amidst an economic recession and political turmoil was Lebanon (Elhage Hassan et al., 11). A qualitative study exploring stakeholders’ perspectives on the Lebanese public health policy regarding menstrual health emphasized the need to decrease the monetary burden by subsidizing menstrual products or by implementing a coupon system. The study also reported the need for proper education on multiple levels, cooperation between key players in the private and public sectors, and encouragement of local production to ensure future sustainability (Elhage Hassan et al.).

When it comes to the United States, low-income menstruating adults were seen to be disproportionately affected by period poverty during the COVID-19 pandemic because the pandemic acted as a barrier to period supplies and an underlying factor for missing work due to the lack of such products (Hunter et al.). Similarly, adults working with U.S-based communities of color revealed tension between school responsibility and family authority in providing menstruation and puberty education in schools, school- and teacher-related delivery challenges, and inadequate and disengaging menstruation and puberty content (Schmitt et al.).

Social, physical, and mental issues imposed by period poverty and encountered by females globally are inhumane and require immediate attention of policymakers and healthcare professionals to attain positive and sustainable menstruation outcomes. Findings from this issue will provide the readers with an overview about the current situation of period poverty in high-income and low-income countries. It will also guide research experts in the design and implementation of interventions seeking to address structural, social, and individual factors among populations impacted by such a phenomenon.
