# Corrigendum: Editorial: Period poverty

**DOI:** 10.3389/frph.2023.1308137

**Published:** 2023-11-20

**Authors:** Lea Sacca, Christine Margaret Markham, Jhumka Gupta, Melissa Peskin

**Affiliations:** ^1^ Charles E. Schmidt College of Medicine, Florida Atlantic University, Boca Raton, FL, United States; ^2^ Center for Health Promotion and Prevention Research, School of Public Health, University of Texas Health Science Center Houston, Houston, TX, United States; ^3^ Department of Global and Community Health, College of Health and Human Services, George Mason University, Fairfax, VA, United States

**Keywords:** period poverty, menstrual equity, women’s rights, sanitary facilities, menstrual hygiene, menstrual health, human rights

A Corrigendum on Editorial: Period poverty By Sacca L, Markham CM, Gupta J and Peskin M (2023). Front. Reprod. Health. 5:1140981. doi: 10.3389/frph.2023.1140981

**Text Correction**

A correction has been made to the third paragraph, second sentence. This sentence previously stated:

“It is estimated that 500 million women in the United States have inadequate access to menstrual products and hygiene facilities (7, 5).”

The corrected sentence appears below:

“It is estimated that 16.9 million menstruating women in the United States live in poverty, two-thirds of which are low-income and food-insecure women who cannot afford basic menstrual products such as pads, tampons, and menstrual products”.

The authors apologize for this error and state that this does not change the scientific conclusions of the article in any way. The original article has been updated.

